# Exploring Links between Genotypes, Phenotypes, and Clinical Predictors of Response to Early Intensive Behavioral Intervention in Autism Spectrum Disorder

**DOI:** 10.3389/fnhum.2013.00567

**Published:** 2013-09-11

**Authors:** Valsamma Eapen, Rudi Črnčec, Amelia Walter

**Affiliations:** ^1^Academic Unit of Child Psychiatry South West Sydney, South Western Sydney Local Health District, Liverpool, NSW, Australia; ^2^School of Psychiatry, University of New South Wales, Sydney, NSW, Australia

**Keywords:** autism spectrum disorder, genotype, phenotype, early intervention, treatment response

## Abstract

Autism spectrum disorder (ASD) is amongst the most familial of psychiatric disorders. Twin and family studies have demonstrated a monozygotic concordance rate of 70–90%, dizygotic concordance of around 10%, and more than a 20-fold increase in risk for first-degree relatives. Despite major advances in the genetics of autism, the relationship between different aspects of the behavioral and cognitive phenotype and their underlying genetic liability is still unclear. This is complicated by the heterogeneity of autism, which exists at both genetic and phenotypic levels. Given this heterogeneity, one method to find homogeneous entities and link these with specific genotypes would be to pursue endophenotypes. Evidence from neuroimaging, eye tracking, and electrophysiology studies supports the hypothesis that, building on genetic vulnerability, ASD emerges from a developmental cascade in which a deficit in attention to social stimuli leads to impaired interactions with primary caregivers. This results in abnormal development of the neurocircuitry responsible for social cognition, which in turn adversely affects later behavioral and functional domains dependent on these early processes, such as language development. Such a model begets a heterogeneous clinical phenotype, and is also supported by studies demonstrating better clinical outcomes with earlier treatment. Treatment response following intensive early behavioral intervention in ASD is also distinctly variable; however, relatively little is known about specific elements of the clinical phenotype that may predict response to current behavioral treatments. This paper overviews the literature regarding genotypes, phenotypes, and predictors of response to behavioral intervention in ASD and presents suggestions for future research to explore linkages between these that would enable better identification of, and increased treatment efficacy for, ASD.

## Genetic Basis of Autism Spectrum Disorder

It has been suggested that autism spectrum disorder (ASD) is one of the most familial of psychiatric disorders, with a heritability of 80%, a monozygotic concordance rate of 70–90%, dizygotic concordance of around 10%, and more than a 20-fold increase in risk for first-degree relatives (Bailey et al., 1995; O’Roak, 2008). Although there have been some significant advances in the recent past (Wang et al., 2009; Pinto et al., 2010), the rate of progress in gene discovery has been modest (Abrahams and Geschwind, 2010). Also, genomic analyses indicate extreme genetic heterogeneity and so far, over 100 genes have been reported in ASD with a conservative estimate of between 380 and 820 loci implicated (Betancur, 2011; Clarke and Eapen, in press), and with considerable overlap with other disorders such as intellectual disability, epilepsy, schizophrenia, and attention deficit hyperactivity disorder (ADHD). These findings suggest that ASD is not a single-gene disorder with Mendelian inheritance but rather a complex disorder resulting from simultaneous genetic variations in multiple genes (Dawson et al., 2002; El-Fishawy, 2010) as well as complex interactions between genetic, epigenetic, and environmental factors (Eapen, 2011).

**Figure 1:**
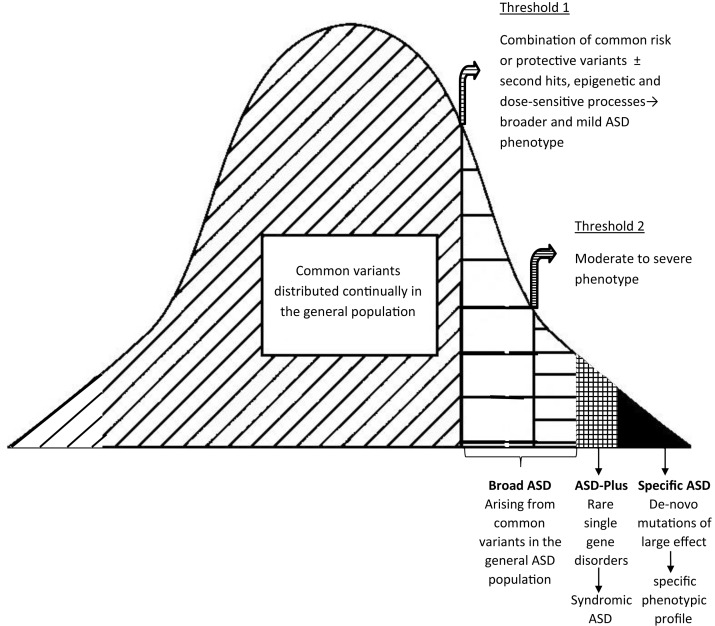
**Genetic and phenotypic heterogeneity in ASD**. Adapted from Eapen (2011).

It has been reported that some of the associated sequence variations noted in ASD are common in the general population although it is unclear as to whether the ASD phenotype results from the involvement of single genes in combination with non-genetic factors, or multiple genes through locus heterogeneity (multiple rare variations in the same gene), or multiple genes through allelic heterogeneity (variations in multiple and different genes). Furthermore, it has been proposed that multiple genes in combination with non-genetic factors may be necessary to result in the ASD phenotype or that ASD may be a collection of rare disorders, that is, a shared phenotype resulting from several different genetic defects. Thus it would seem that there are at least three major pathogenetic processes (Eapen, 2011) resulting in three different subgroups: (1) ASD-Plus group or Syndromic ASD resulting from rare single-gene disorders where ASD is a behavioral phenotype of the associated disorder; (2) Broad ASD group resulting from common variants distributed continually in the general population but following a gene-environment diathesis model, when it passes the first threshold (threshold 1) due to other gene or environmental additive effects or “second hits” including epigenetic and dose-sensitive processes, it results in the broader and mild ASD phenotype which may be observed in other family members of affected individuals and when it passes a second threshold (threshold 2) it results in a moderate to severe ASD phenotype that is clinically significant; and (3) specific ASD group due to “*de novo* mutations” of large effect resulting in ASD presentations but carrying unique phenotypic profiles based on the specific site and nature of the *de novo* mutation (see Figure 1).

## Broad Autism Phenotype

The term “broad autism phenotype” (BAP) refers to the presence of subclinical levels of ASD symptoms among individuals who do not meet criteria for a diagnosis of ASD (Bolton et al., 1994; Piven and Palmer, 1999). BAP characteristics correspond to the primary features of ASD, including traits that are social, such as socially reticent or inappropriate behavior, or non-social, such as rigidity and ritualistic or repetitive behaviors (Losh et al., 2009). Twin and family studies have shown that genetic liability to autism is expressed in unaffected relatives of people with ASD through features that are milder but qualitatively similar to the defining characteristics of ASD, including social abnormalities, communication impairments, and repetitive behaviors (Bailey et al., 1998; Goussé et al., 2002; Losh et al., 2009). Previous studies suggest that around 25% of first-degree relatives of children with ASD show impairment in one of the three diagnostic domains for ASD: sociability, communication, and cognitive or behavioral flexibility (for a review, see Goussé et al., 2002). Bailey et al. (1998) conclude that the BAP features observed in relatives of individuals with ASD appear to have a genetic rather than environmental basis.

While early work on the BAP focused on examining ASD-related traits in first and second degree relatives of individuals with ASD (for a review, see Bailey et al., 1998), subsequent studies have demonstrated that the characteristics comprising the BAP exist within the general population as well (Baron-Cohen et al., 2001; Constantino and Todd, 2003, 2005). Features of ASD that have been found to be continuously distributed within the general population include restricted interests (Baron-Cohen et al., 2001), atypical visuospatial and cognitive performance (Grinter et al., 2009; Stewart et al., 2009; Richmond et al., 2013), abnormal speech perception (Stewart and Ota, 2008), reduced gaze reciprocity (Chen and Yoon, 2011), an impaired ability to recognize affect from facial expressions and body language (Ingersoll, 2010b), and reductions in social skill and social-cognitive ability (Sasson et al., 2012). These findings may be consistent with the suggestion that some of the genetic sequence variations found in ASD are common in the general population.

## Heterogeneity of Autism

Despite major advances in the genetics of ASD, the relationship between different aspects of the behavioral and cognitive phenotype of ASD and their underlying genetic liability is still unclear (Bailey et al., 1998; Klin et al., 2002). This is complicated by the heterogeneity of ASD, which exists at both genetic and phenotypic levels (Charman et al., 2011). Further, it has been suggested that there may be gender dependent differences in the ASD phenotype (Eapen, 2011). For example, Lai et al. (2012) observed that while performance in the social-cognitive domain was equally impaired in male and female adults with ASD, in the specific non-social-cognitive domains of attention to detail and dexterity involving executive function, there were differences based on gender. Losh et al. (2009) argue that the BAP may provide an important complementary approach for detecting the genes involved in ASD by narrowing the highly heterogeneous phenotype of an ASD diagnosis to particular features that are likely to be more conducive to genetic investigation (Wheelwright et al., 2010; Spencer et al., 2011, 2012a,b; Sucksmith et al., 2012).

Due to its heterogeneity, ASD is no longer viewed as a narrowly defined, categorical disorder, but instead as a spectrum of conditions that affect individuals differently (Wing, 1996). Some researchers have suggested that there are probably many “autisms” with different underlying biological processes and developmental pathways (Elsabbagh, 2012). The term ASD is now commonly used to describe a range of neurodevelopmental conditions that show considerable phenotypic heterogeneity at any one age and across development, and that are likely to differ in underlying etiology (Geschwind and Levitt, 2007). However, they all generally share a primary impairment in social relatedness and reciprocity, an “insistence on sameness,” and impairments in the use of language for communication, which is in keeping with Kanner’s (1943) description of classically “autistic” children.

It is noteworthy that genetic heterogeneity leads to clinical heterogeneity. For example, similar or identical mutations can result in very broad phenotypic variations as is evident from studies investigating endophenotypes exhibited by patients expressing mutations in the CNTNAP2 gene (Eapen, 2011). Such studies demonstrate a role for CNTNAP2 in schizophrenia, epilepsy, Tourette’s syndrome, and obsessive compulsive disorder (Verkerk et al., 2003; Friedman et al., 2008). Alternatively, ASD cases resulting from different genetic lesions can have clinically distinct presentations (Bruining et al., 2010). However, such distinct phenotypic presentations are masked by the limitations of diagnostic categories. Therefore, future studies exploring risk alleles should examine homogenous and heritable endophenotypic traits rather than diagnostic groups. Thus, given the significant genotype to phenotype heterogeneity, one method to find homogeneous entities and link these with specific genotypes would be to pursue endophenotypes.

## Endophenotypes in ASD

Neurocognitive profiles and neurophysiological changes observed using neuroimaging, eye tracking, and electrophysiological techniques are commonly reported in individuals with ASD. Studies of head circumference and imaging studies of brain morphometry have found evidence of increased brain growth beginning within the first year of life (Courchesne et al., 2005), while functional brain imaging in older children and adults has shown abnormal patterns of interactions between brain regions, possibly related to aberrant connections being laid down during earlier stages of development (Courchesne et al., 2011). One model relating these early abnormalities in brain development to the characteristic socio-communicative impairments has hypothesized that early low-level deficits in recognition and orientation toward social stimuli lead to a lack of social engagement with primary caregivers during infancy, resulting in decreased exposure to the reciprocal social interactions critical for healthy development of brain circuits responsible for normal social behavior (Dawson, 2008).

Basic, low-level impairments of social attention and reciprocity are thought to relate to the socio-communicative impairments characteristic of ASD and are evident in children with ASD from as early as the first year of life. For example, home videos of 12-month-olds later diagnosed with ASD demonstrate reduced visual attention to people and failure to respond to vocal approaches (Werner et al., 2000; Osterling et al., 2002; Werner and Dawson, 2005), while other studies have shown poor verbal imitation (Sallows and Graupner, 2005). Prospective studies of children at high risk of ASD show similar results (Nadig et al., 2007). Young children with ASD also show a lack of joint attention and failure to coordinate attention and share their experiences with caregivers (Charman, 2003). Researchers using preferential looking techniques have identified a reduction in autistic toddlers’ preference for viewing biological motion (Klin et al., 2009) and hearing the human voice (e.g., Klin, 1991; Dawson et al., 1998, 2004).

Similarly, eye gaze abnormalities have been described as indicative of later development of ASD (Bedford et al., 2012; Elsabbagh et al., 2012). Using eye tracking technology, Jones et al. (2008) found that 2-year-olds with ASD lacked the normal bias to attend to the eyes when watching videos of people, replicating earlier studies with autistic adolescents (Klin et al., 2003; see also Norbury et al., 2009) and confirming clinical reports of reduced eye contact in ASD (Zwaigenbaum et al., 2005). Psychophysical evidence suggests that differences in spatial localization between individuals with ASD and controls begins at an early cortical stage of visual processing (Latham et al., 2013). Further evidence comes from electrophysiology. Pre-school and school-aged children with ASD produce atypical cortical event-related potentials (ERPs) in response to deviations in streams of speech stimuli, despite normal responses to deviants in streams of non-speech stimuli (Kuhl et al., 2005; Lepistö et al., 2005; Ceponiene et al., 2005; Whitehouse and Bishop, 2008). Kuhl et al. (2013) compared brain responses to word stimuli between typically developing children and children with ASD, categorized into two groups according to the severity of their social symptoms. They found that the brain activity of children with ASD with less severe social symptoms resembled that of the typically developing controls, while children with ASD with more severe social symptoms showed a clearly atypical brain response. Furthermore, the ERP response among children with ASD at time 1 (when they were 2 years old) was found to predict receptive language, cognitive ability, and adaptive behaviors at two follow-up time points, when the children were 4 and 6 years old (Kuhl et al., 2013). Similarly, school-aged children are reported to show abnormal brainstem evoked responses (ABR) to trains of speech stimuli but not click sounds (Russo et al., 2009), and these abnormalities are linked to clinical assessments of language abilities.

Recognition of facial emotions has also been found to be impaired in children and adults with ASD compared to controls (Sucksmith et al., 2012; Oerlemans et al., 2013). The reduced activation in brain regions associated with facial processing in people with ASD relative to control subjects has been shown to be correlated with the clinical severity of their impairment in reciprocal social interaction (Spencer et al., 2012b). Finally, studies using electromyography (EMG) to measure facial muscle activity have shown a reduction or delay in the normal tendency to (subconsciously) mimic emotional expressions when viewing pictures of faces (McIntosh et al., 2006; Oberman et al., 2009).

Further, it is widely argued that many of the symptoms of ASD are caused by aberrant neural connectivity (e.g., Brock et al., 2002; Geschwind and Levitt, 2007), including specific findings such as reduced functional connectivity within and between “social” resting state networks in ASD (von dem Hagen et al., 2013) as well as significantly increased gray matter volume in the anterior temporal and dorsolateral prefrontal regions and significant reductions in the occipital and medial parietal regions compared with controls (Ecker et al., 2012). These findings imply that a key component of behavioral intervention may be to compensate for such early deficits and that behavioral intervention should occur as early as possible to normalize the developmental trajectory and avoid downstream effects. Thus baseline performance on neurocognitive responses to socially relevant stimuli might predict the magnitude of clinical and cognitive improvement following behavioral intervention. Recent research suggests that early behavioral intervention may be associated with normalized brain activity in young children with ASD. Previous studies have demonstrated that children receiving the Early Start Denver Model (ESDM), a behavioral intervention for children with ASD, showed gains in IQ and adaptive behavior and decreases in ASD-specific symptoms after intervention (Dawson et al., 2010; Eapen et al., 2013; Vivanti et al., 2013). In a recent study, Dawson et al. (2012) found that typically developing children and children with ASD who had been treated with the ESDM showed more cortical activation and allotted greater attentional and cognitive resources to social stimuli than to non-social stimuli, while children with ASD who had received community-based behavioral intervention showed the reverse pattern.

## Behavioral and Cognitive Phenotypes in ASD

There are increasing efforts to determine and refine subtypes within the ASD behavioral phenotype (e.g., Ingram et al., 2008; Munson et al., 2008a,b; Frazier et al., 2010), with contemporary studies using large samples and sophisticated statistical approaches such as taxometric and latent variable models. To date, however, few distinct behavioral subtypes have been identified, and none is yet well replicated – frustrating efforts to “carve nature at the joints.” Ingram et al. (2008) provided the first taxometric analysis of ASD and sought to test putative ASD subgrouping paradigms based on seven phenotypes which vary within the ASD population: social interaction/communication, intelligence, adaptive functioning, insistence on sameness, repetitive sensory motor actions, language acquisition, and essential/complex physical phenotype. The “complex” physical phenotype was defined according to the presence of physical dysmorphology and/or microcephaly, indicating some abnormality of early morphogenesis, whereas the “essential” physical phenotype referred to the remainder of individuals with ASD without these features (Miles et al., 2005). The authors indicated that valid subgroups could be constructed using the social interaction/communication, intelligence, and essential/complex paradigms, whereas the other phenotypes were found to exhibit results consistent with a dimensional structure. Given intelligence is consistently described as one of the primary aspects of heterogeneity in ASD, Munson et al. (2008a) sought to explore whether there were distinct ASD subtypes based upon IQ. Four latent classes were ultimately identified that represented different levels of intellectual functioning as well as different patterns of relative verbal versus non-verbal abilities. Moreover, group membership was related to adaptive functioning and social impairment, above and beyond the direct relationship of verbal and non-verbal IQ (Munson et al., 2008a). In a different study, Munson et al. (2008b) reported that specific aspects of neurocognitive functioning appear to be important predictors of developmental variability during the pre-school years in children with ASD. In particular, learning of reward associations and imitation from memory and novelty preference were significantly related to Vineland socialization and communication growth rates above and beyond non-verbal problem solving ability. A review of factor analytic studies showed that, of the seven studies included, six found evidence for multiple factors underlying autistic features (Mandy and Skuse, 2008). The majority of studies reported at least one social-communication factor and all but one also reported at least one distinct non-social factor comprising repetitive interests, behaviors and activities, however, the total number of factors reported varied.

In a large scale study employing taxometric and latent variable models, Frazier et al. (2010) concluded that the available literature and study results implied a categorical model of ASD, with two to three subdimensions – social communication, repetitive/perseverative behavior, and possibly social motivation – best reflecting the structure of ASD symptoms. Related work by the same group yielded similar results and provided broad support for DSM5 ASD criteria (Frazier et al., 2012). This finding is somewhat at odds with the related body of literature that has concluded that ASDs represent the severe end of a quantitative trait or continuum of social behavior, and the differing conclusions may reflect differing theoretical and statistical approaches. It is of course also possible that both viewpoints are correct and that categorical and dimensional aspects of ASD symptoms should be considered in the conceptualization of ASD.

There is increasing momentum within the literature to conceive of the core ASD symptomatology as distinct, or “fractionable.” That is, that while the core features may regularly co-occur, these features may have distinct causes at genetic, cognitive, and neural levels. In their seminal review paper, Happe et al. (2006) argue that “*it is time to give up on the search for a monolithic cause or explanation for the three core aspects of autism*” (p. 1219). This claim was based in part on Ronald and colleagues’ work on a large UK general population twin sample which found that correlations between continuous measures of social, communication and repetitive behavior were lower than expected (Happe et al., 2006). Happe et al. describe several implications following from their thesis, including that at the behavioral level each aspect of the ASD behavioral triad needs to be assessed separately rather than using global rating scales. The authors also claim that “*heterogeneity in ASD, on our account, is not simply due to noise or the complex unfolding of development, but is an unavoidable consequence of variation along at least three largely independent (although of course interacting) dimensions of impairment*” (p. 1220).

A large number of studies have also explored the ASD cognitive phenotype and a number of cognitive models of ASD have been proposed over time. These include the theory of mind account (Baron-Cohen et al., 1985); the executive dysfunction account (Ozonoff et al., 1991); the weak central coherence account (Happe and Frith, 2006); the enhanced perceptual functioning account (Mottron et al., 2006); the theory of reduced generalization and enhanced discrimination ability (Plaisted, 2001); and the empathizing – systematizing theory (Baron-Cohen, 2010; Grove et al., 2013). Each of these cognitive characteristics has been successfully linked to specific aspects of the ASD behavioral phenotype (Taylor et al., 2012) although none would appear to provide a parsimonious account of features observed in ASD. Charman et al. (2011) provide an excellent review of studies in this area and also a compelling account of the potential benefits of articulating ASD cognitive phenotypes with respect to advancing both treatment and genetic research. Charman also highlights the challenges involved in conducting high quality research in this area from statistical and methodological perspectives.

## Variability in and Predictors of Response to Behavioral Treatment in ASD

The heterogeneity of ASD may also underlie the variability in response to treatment that is observed among individuals with ASD. Meta-analyses conducted in recent years have tended to conclude that Early Intensive Behavioral Intervention (EIBI), incorporating the principles of applied behavior analysis (ABA), is the treatment of choice for young children with ASD (Vismara and Rogers, 2010; Reichow, 2012), and that superior outcomes are associated with entry into EIBI at the earliest possible age (Granpeesheh et al., 2009; Wallace and Rogers, 2010). Despite the efficacy of EIBI for some children, there is tremendous variation in treatment response in ASD, with other children who receive EIBI failing to have a dramatic response (Dawson et al., 2002). A systematic review of controlled studies of EIBI showed that, while EIBI resulted in improved outcomes for children with ASD compared to comparison groups at a group level, there was marked variability in outcome at an individual level (Howlin et al., 2009). This differential response to treatment is common across all of the evidence-based approaches for treatment of ASD, with up to 50% of children showing substantial positive gains, and the other 50% making variable progress, some with extremely limited skills development (Stahmer et al., 2011).

Therefore, research aimed at methods of individualizing treatment is important. Such research requires an understanding of the pre-treatment characteristics associated with differential response to treatment, including child and family variables, and how specific behavioral intervention techniques address each of these characteristics (Stahmer et al., 2011). The goal of this line of research is to allow treatments to be tailored to individual children and thereby increase the overall rate of positive outcomes for children with ASD (Stahmer et al., 2011). In a recent systematic review of EIBI for ASD, however, Warren et al. (2011) concluded that the ability to predict children’s response to treatment and outcome was very limited and warranted further investigation. The genetic and phenotypic heterogeneity inherent in ASD may also imply that no single EIBI can be universally effective and that, in a sense, many nuanced treatment approaches may ultimately be required for the many autisms in existence.

Nonetheless, available evidence indicates that a number of pre-treatment factors may be associated with response to treatment across various EIBI models. These include overall IQ (McEachin et al., 1993; Harris and Handleman, 2000; Eldevik et al., 2006; Magiati et al., 2007; Remington et al., 2007; Perry et al., 2011), language and communication abilities (Sallows and Graupner, 2005; Eldevik et al., 2006; Eikeseth et al., 2007; Magiati et al., 2007; Remington et al., 2007), adaptive skills (Remington et al., 2007; Makrygianni and Reed, 2010; Flanagan et al., 2012), imitation (Sallows and Graupner, 2005; Vivanti et al., 2013), play skills (Kasari et al., 2008, 2012; Ingersoll, 2010a), joint attention (Yoder and Stone, 2006; Kasari et al., 2008), interest in objects (Yoder and Stone, 2006; Schreibman et al., 2009; Carter et al., 2011), functional use of objects (Vivanti et al., 2013), symptom severity (Smith et al., 2000; Sallows and Graupner, 2005; Remington et al., 2007; Vivanti et al., 2013), and younger age (Harris and Handleman, 2000; Perry et al., 2011). Some studies, however, have failed to find relationships between these factors and treatment response. For example, Eldevik et al. (2006) found that age at intake was not a predictor of children’s response to a low-intensity behavioral treatment, while Sallows and Graupner (2005) found that initial IQ did not predict children’s response to an intensive behavioral intervention. Furthermore, the direction of relationships between these pre-treatment factors and intervention response is sometimes inconsistent. For example, Remington et al. (2007) found that higher ASD symptom scores at intake were associated with improved EIBI outcomes, while Smith et al. (2000) found that children with milder symptoms (i.e., a diagnosis of Pervasive Developmental Disorder-NOS) tended to have a better response to EIBI than children with more severe symptoms (i.e., a diagnosis of ASD).

## Increasing Treatment Efficacy for ASD by Identifying Individual Differences

Given the heterogeneity of ASD, it is likely that a personalized medicine approach, considering individual differences in etiologic and phenotypic characteristics, would result in increased treatment efficacy (Perrin et al., 2012). Georgiades et al. (2013) suggest that, rather than conducting studies that compare individuals with a diagnosis of ASD with typically developing individuals, future research should focus on understanding the meaning of individual and subgroup differences within the autism spectrum on treatment outcomes. The identification of such differences, and an understanding of how these might impact on response to treatment, has implications for the ability to individually tailor treatment programs and thereby improve their effectiveness.

As an example, this type of approach has been found to be useful in explaining differences in presentation and treatment response in children with diagnoses of oppositional defiant disorder and conduct disorder. There is increasing support for the subtyping of childhood conduct problems based on whether children exhibit high versus low levels of callous-unemotional (CU) traits, such as a lack of guilt and empathy (Hawes et al., 2013). Research suggests that the conduct problems of children with high levels of CU traits are more severe and less responsive to established psychological interventions than those of children without CU traits (Hawes and Dadds, 2005; Waschbusch et al., 2007). This has allowed research into interventions that may contribute to reductions in the CU traits of young children (Hawes and Dadds, 2007; McDonald et al., 2011). Furthermore, articulating these phenotypic differences has contributed to a better understanding of the etiology of conduct disorder (e.g., Viding et al., 2007).

**Figure 2:**
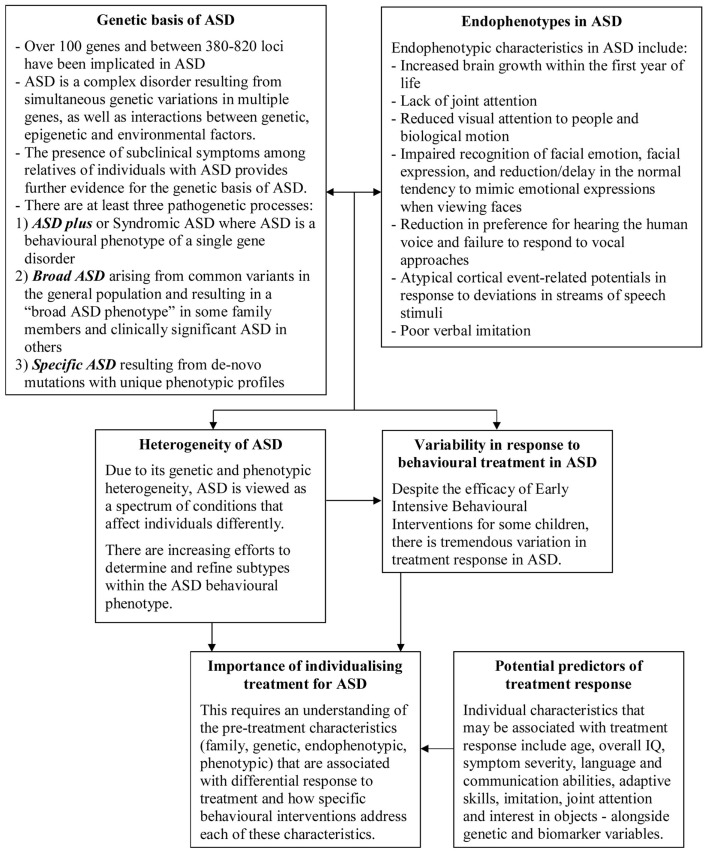
**Summary of key points and links between genotypes, endophenotypes, and clinical predictors of response to behavioral intervention in ASD**.

One method to potentially identify subgroups among children with ASD would be to investigate phenotypic characteristics that may predict response to treatment, which has important implications for guiding choice of treatment. Furthermore, using a longitudinal design, it would be beneficial to compare the developmental trajectory of young children with ASD receiving a standardized treatment with those in “waitlist” conditions as well as with healthy control groups. This would provide a significant contribution to the sparse body of knowledge about developmental changes in brain function during this period of development. Even more importantly, determining which characteristics correspond with observable changes in the treatment group would allow us to identify specific individual characteristics and relevant biomarkers sensitive to behavioral intervention, with implications for assessing response to intervention in clinical and research settings (see Figure 2). This would also reveal whether and which variations in the baseline measures of brain function predict response to treatment. Given the significant investment represented by EIBI programs, their overall utility could be greatly enhanced by determining whether there are measurable characteristics at baseline capable of predicting response. Finally, if such phenotypic predictors were established, this could further our understanding of the genetic basis of ASD, by allowing future research to attempt to link these predictors to specific underlying genetic causes. The task ahead is certainly great, but also tantalizing with respect to the potential refinements in our understanding and also benefits to affected individuals that are possible.

## Conflict of Interest Statement

The authors declare that the research was conducted in the absence of any commercial or financial relationships that could be construed as a potential conflict of interest.
